# Anti-MET Antibody Therapies in Non-Small-Cell Lung Cancer: Current Progress and Future Directions

**DOI:** 10.3390/antib13040088

**Published:** 2024-10-18

**Authors:** Kinsley Wang, Robert Hsu

**Affiliations:** 1 Department of Medicine, University of Arizona College of Medicine—Phoenix, Phoenix, AZ 85004, USA; kinsleywang@uarizona.edu; 2 Department of Medicine, Division of Medical Oncology, University of Southern California Norris Comprehensive Cancer Center, Los Angeles, CA 90033, USA

**Keywords:** MET (mesenchymal–epithelial transition factor), NSCLC (non-small-cell lung cancer), EGFR-mutated NSCLC, targeted therapies, amivantamab

## Abstract

**Background/Objectives:** Non-small-cell lung cancer (NSCLC) remains a leading cause of cancer mortality globally, though advances in targeted therapies have improved treatment outcomes. The mesenchymal–epithelial transition (*MET*) gene plays a significant role in NSCLC, often through protein overexpression, exon 14 skipping mutations, and gene amplification, many of which arise as resistance mechanisms to other oncogenic drivers like epidermal growth factor receptor (*EGFR*) mutations. This review examines the development and clinical efficacy of anti-MET antibody therapies. **Methods:** A comprehensive literature search was conducted using major medical databases looking at key relevant studies on anti-MET antibody studies. Both authors reviewed the literature, assessed study quality, and interpreted the results from each study. **Results:** Amivantamab, a bispecific EGFR/MET antibody was approved to treat EGFR exon 20 insertion and now has recently been extended to target classical EGFR mutations with progression on osimertinib. Other important anti-MET targeted therapies in development include antibody drug conjugates such as telisotuzumab vedotin, REGN5093-M114, and AZD9592 and emibetuzumab, which is a humanized immunoglobulin G4 monoclonal bivalent MET antibody. **Conclusions:** MET plays a significant role in NSCLC and amivantamab along with other anti-MET targeted therapies play a role in directly targeting MET and addressing acquired resistance to oncogenic drivers. Future research should focus on developing novel MET antibody drugs and exploring new therapeutic combinations to enhance treatment efficacy and overcome resistance in NSCLC. Refining biomarker-driven approaches to ensure precise patient selection is also critical to optimizing treatment outcomes.

## 1. Introduction

The mesenchymal–epithelial transition (*MET*) gene encodes the MET receptor, a tyrosine kinase that drives key processes in cancer, including cell growth, survival, migration, and invasion by activating signal transduction pathways, including the RAS-MAPK cascade, PI3K-AKT pathway, and STAT pathways. MET’s interaction with its ligand, hepatocyte growth factor/scatter factor (HGF/SF), is particularly critical in promoting the epithelial to mesenchymal transition (EMT), which enhances tumor cell proliferation, motility, and anchorage-independent growth [1]. Additional MET properties include initiating early coagulation processes to form fibrin structures that aid clonal expansion, maintaining tumorigenic potential amidst therapeutic and immune challenges, and facilitating systemic dissemination, supporting its contribution to tumor growth and metastasis. In epithelial cells, MET-HGF/SF interactions activate downstream proteins like Gab1, initiating signaling cascades, such as Shp2, PI3K, and Crk, which induce cellular responses necessary for normal and tumor cell growth [2].

This extensive signaling network underpins MET’s role in driving invasive growth, which explains the ability of cancer cells to both move and proliferate in response to growth factors [3,4]. Invasive growth, facilitated by EMT, is characterized by cellular plasticity and the dual activation of cell stemness and dissemination sustained over extended periods [5,6]. MET’s ability to sustain invasive growth under diverse conditions explains its contribution to metastasis and therapeutic resistance. Furthermore, MET facilitates angiogenesis by inducing pro-angiogenic factors such as Vascular Endothelial Growth Factor A (*VEGFA*), and inhibiting angiogenic suppressors, such as TSP1. Given the MET gene’s broad involvement in tumor progression and metastasis, it is a pivotal target in cancer therapy, particularly in addressing treatment resistance and enhancing therapeutic outcomes.

Non-small-cell lung cancer (NSCLC) is the most prevalent form of lung cancer and remains a leading cause of cancer-related mortality worldwide [7]. The aggressive nature of NSCLC, characterized by its high metastatic potential and frequent resistance to conventional therapies, underscores the importance of understanding its molecular drivers to develop effective treatments. Among these drivers, the dysregulation of MET plays a critical role in promoting tumor progression and resistance to targeted therapies. Consequently, MET has emerged as a key therapeutic target in NSCLC, particularly in addressing mechanisms of resistance that hinder long-term treatment efficacy.

**Figure 1:**
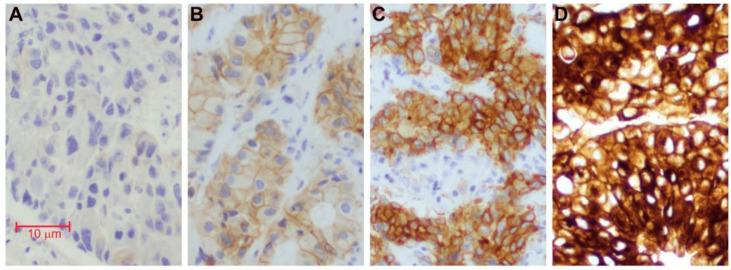
Examples of MET expression level by immunohistochemistry: (**A**) absent (0+); (**B**) weak (1+); (**C**) moderate (2+); (**D**) strong (3+). Reproduced from DOI: 10.3390/cancers14102433 (2022), MDPI [14].

In NSCLC, the three main mechanisms of MET dysregulation are protein overexpression, exon 14 skipping (METex14) mutations, and gene amplification. METex14 mutation impairs MET protein receptor degradation, leading to the sustained activation of MET signaling, which promotes cell proliferation and tumor growth [8]. These mutations are found in about 2% to 4% of advanced NSCLC, predominantly occurring in adenocarcinoma and pulmonary sarcomatoid carcinoma subtypes [9]. Recent trials also suggest a higher prevalence of METex14 mutation in squamous NSCLC, at 9% prevalence [10]. Currently, METex14 is identified through DNA next-generation sequencing platforms, Sanger sequencing, or RNA-based assays. MET amplification involves either polysomy or the amplification of the MET gene on chromosome 7. Amplification, characterized by an increased MET-to-centromere 7 ratio, tends to result in oncogene addiction, observable in approximately 1% to 5% of untreated NSCLC cases [11,12]. Such amplifications can occur as primary driver alterations or as mechanisms of acquired resistance after treatment with tyrosine kinase inhibitors (TKIs). Additionally, MET overexpression, detected by immunohistochemistry (IHC), occurs in 35% to 72% of NSCLC cases and correlates with poorer clinical outcomes [13]. Overexpression is classified as either absent, weak, moderate, or strong (Figure 1). MET overexpression may co-occur with MET exon 14 skipping mutations or MET amplification, further complicating diagnosis and treatment.

While MET dysregulation is a critical driver of NSCLC, EGFR mutations also play a significant role. EGFR mutations are the most common mutation in NSCLC, with incidence in 30–40% of NSCLCs in Asian patients and 10% in Caucasian patients [15]. The most prevalent activating EGFR mutations are exon 19 deletions (EGFRdel19) and point mutations in exon 21 (EGFR L858R), which account for 45% and 40% of all EGFR mutations, respectively [16,17,18]. First-generation EGFR TKIs, gefitinib and erlotinib, initially control NSCLC harboring these mutations [18]. However, treatment resistance usually develops after about one year [19]. In 50–60% of cases, this acquired resistance is driven by secondary EGFR mutations, most commonly T790M [20]. Third-generation EGFR TKIs, such as osimertinib, are designed to specifically target the T790M mutant form of EGFR and are highly effective [21]. Nonetheless, resistance to third-generation EGFR TKIs commonly occurs, driven by additional mutations in EGFR or the activation of alternative pathways such as MET amplification [22,23,24,25,26]. This underscores the growing interest in targeting MET as a therapeutic strategy for overcoming resistance to EGFR TKIs.

Given the significant role of MET in NSCLC, several MET-targeting therapies have been developed and approved, with more in various stages of development. Traditionally, most MET-targeting therapies have been TKIs, such as crizotinib, capmatinib, tepotinib, savolitinib, and glumetinib. Crizotinib was the first anti-MET TKI that reported significant activity in the PROFILE 1001 trial [27]. Crizotinib, in combination with tepotinib, was approved by the Federal Drug Administration (FDA) for MET exon 14 skipping mutations [28]. More recent research has shifted focus to novel antibody-based therapeutics, which will be discussed within this paper. A list of MET antibody drugs and their clinical results are listed in Table 1 and will be discussed throughout this review.

## 2. Amivantamab

Amivantamab is a bispecific EGFR/MET antibody that inhibits ligand binding to the EGFR and MET receptors, preventing their dimerization and subsequent downstream signal transduction. Additionally, it facilitates antibody-dependent cellular cytotoxicity (ADCC) and promotes the downregulation of cell surface proteins. Amivantamab was originally evaluated for EGFR exon 20 insertion (Exon20ins) mutations. Patients with Exon20ins, which represents 4% to 12% of all EGFR mutations, are generally insensitive to approved EGFR TKIs like osimertinib and are associated with poor prognosis. Based on the phase I/IB CHRYSALIS trial (NCT02609776), where amivantamab was used to treat Exon20ins (*n* = 81) in patients that had progressed on chemotherapy, the objective response rate (ORR) was 40%, with a median duration of response (mDOR) of 11.1 months [29]. Median progression-free survival (mPFS) was 8.3 months. Due to its efficacy, durability of response, and manageable safety profile, the FDA designated amivantamab as a breakthrough therapy for treating EGFR exon 20 insertion mutations.

In building upon successful antitumor activity in the phase 1 trial, an additional phase 3 trial PAPILLION (NCT04538664) evaluated amivantamab plus chemotherapy versus standard chemotherapy alone as a first-line treatment for advanced NSCLC with EGFR Exon20ins mutation [32]. The amivantamab–chemotherapy cohort (*n* = 153) had an mPFS of 11.4 months, which was significantly higher than 6.7 months for chemotherapy alone (*n* = 155). The ORR for amivantamab–chemotherapy was 73%, which was greater than chemotherapy at 47%. Amivantamab–chemotherapy was concluded to result in superior efficacy and duration of response as a first-line drug.

Amivantamab and platinum chemotherapy (carboplatin and pemetrexed) was also evaluated in combination with lazertinib, a brain-penetrant third-generation EGFR TKI, in the LACP cohort of the CHRYSALIS-2 trial (NCT04077463) [33]. This cohort enrolled EGFRm NSCLC with disease progression on or after treatment with EGFR TKI (*n* = 20) and reported an mPFS of 14.0 months and an ORR of 50%.

The same CHRYSALIS trial (NCT02609776) also evaluated amivantamab plus lazertinib in EGFRm NSCLC that had disease progression on third-generation TKI monotherapy (osimertinib-relapsed) but was chemotherapy naïve [30]. The drug combination had an ORR of 36%, with an mDOR of 9.6 months and an mPFS of 4.9 months. The results suggested that amivantamab’s dual action on EGFR and MET could enhance the initial anti-EGFR effects of lazertinib. The dual blockade effect of amivantamab may also postpone the development of resistance, which commonly arises through EGFR secondary mutations and MET activation as alternative signaling pathways.

CHRYSALIS-2, a phase 1/1b trial (NCT04077463), evaluated the same drug combination of amivantamab plus lazertinib in the post-platinum chemotherapy/post-osimertinib setting for patients with advanced EGFRm NSCLC [34]. One arm targeted patients with EGFR Exon19del or L858R NSCLC with progression on osimertinib and chemotherapy (*n* = 162; 50 efficacy evaluable). For the cohort, the ORR was 36%, and the clinical benefit rate (CBR) was 58%. The mDOR was not reached based on the BICR, but seven responders (39%) were reported to have achieved a DOR lasting ≥6 months.

Atypical EGFR mutations span exons 18 to 21 and account for 10–15% of EGFRm NSCLC. An expansion cohort C of CHRYSALIS-2 specifically examined amivantamab plus lazertinib in atypical non-exon 20 insertion EGFR mutations (*n* = 40), with exon 19 G719X mutations counting for 60%, in both the post-afatinib (EGFR TKI) and treatment-naïve population [35]. The best partial response (PR) result was observed in 60% of patients. The ORR was 56.7% (*n* = 30 evaluated).

Phase 3 MARIPOSA (NCT04487080) continued testing the same drug combination but instead as a first-line treatment: It evaluated amivantamab plus lazertinib (*n* = 429) versus osimertinib (*n* = 429) in the first-line setting for EGFRm NSCLC [37]. The ORR was similar for amivantamab–lazertinib versus osimertinib at 86% and 85%, respectively. However, amivantamab–lazertinib demonstrated significantly higher mDOR and mPFS values: The mDOR among confirmed responders was 25.8 months for amivantamab–lazertinib versus 16.8 months for osimertinib; the mPFS was 23.7 months versus 16.6 months, respectively. However, EGFR- and MET-related AEs were overall higher for amivantamab–lazertinib than osimertinib. The significant improvement in PFS and DOR resulted in FDA approval of amivantamab and lazertinib as first-line treatment for locally advanced or metastatic NSCLC with EGFR exon 19 deletion or exon 21 L858R substitution mutation in 2024 [54].

MARIPOSA-2 (NCT04988295), a phase 3 trial, built upon previous drug combinations evaluated in phase 1 studies by evaluating amivantamab–lazertinib–chemotherapy (ami-laz-CT) versus amivantamab–chemotherapy (ami-CT) versus chemotherapy alone in EGFRm NSCLC patients with disease progression on or after osimertinib as the most recent line of treatment [38]. Consistent PFS was significantly higher for ami-laz-CT and ami-CT than for chemotherapy alone, at 8.2 and 8.3 months versus 4.2 months, respectively. Intracranial mPFS had a similar pattern, with 12.8 and 12.5 versus 8.3 months, respectively. Similarly, the ORR was notably higher for ami-laz-CT and ami-CT, at 63% and 64%, respectively, compared to chemotherapy at 36%. However, ami-CT did report lower rates of hematological AEs compared to ami-laz-CT. Overall, ami-laz-CT and ami-CT both improved PFS and intracranial PFS compared to chemotherapy after osimertinib progression, and longer follow-up was needed to continue monitoring efficacy and AEs.

PALOMA-3 (NCT05388669) is a phase 3 trial that evaluated subcutaneous (*n* = 206) compared to intravenous (*n* = 212) amivantamab, both combined with lazertinib, after progression following osimertinib and platinum-based chemotherapy in EGFRm advanced NSCLC, with the goals of improving tolerability and reducing administration time in subcutaneous route [36]. Pharmacokinetically, the subcutaneous route was noninferior to the intravenous route, with a geometric mean ratio for trough concentrations of 1.15 and a cycle-2 area under the curve of 1.03. The mPFS was 6.1 months in the subcutaneous group and 4.3 months in the intravenous group; the ORR was 30% and 33%, respectively. Regarding AEs, the subcutaneous cohort had a lower incidence of infusion-related reactions (13% vs. 66%) and venous thromboembolism (9% vs. 14%). More patients also considered subcutaneous treatment convenient (85% vs. 35%). Overall, subcutaneous amivantamab–lazertinib was noninferior to intravenous administration, with a consistent safety profile.

In addition to treating EGFRm NSCLC, amivantamab was used to treat METex14 mutation in a MET-2 cohort (*n* = 36, 6 treatment-naïve) of the CHRYSALIS trial discussed above (NCT02609776) [31]. For this population, the ORR was 33.3%, with an mPFS of 6.7 months (8.3 vs. 4.2 months for patients without versus with previous MET inhibitor treatment). Enrollment into the MET-2 cohort is ongoing due to promising results. Another ongoing phase 2 trial, METalmark, is combining amivantamab with capmatinib (specific MET TKI) (NCT05488314).

Amivantamab has a spectrum of infusion reactions and toxicities. Infusion-related reactions were common, occurring in 66% of patients, although the incidence was decreased with the subcutaneous administration of amivantamab to 13% [36]. Hematological common adverse effects (AEs) were neutropenia and thrombocytopenia (69% and 60%, respectively for amivantamab–lazertinib–chemotherapy) [38]. Dermatological toxicities were also notable and typically associated with EGFR inhibitors. Common dermatological AEs were acneiform eruption and pruritis. Other patients experienced severe, atypical acneiform eruptions, particularly on the scalp, with symptoms including thick crusts, purulent exudate, and boggy scalp [55]. Widespread inflammatory papules, scaly plaques, and neck dermatitis flared during infusions. Additionally, five patients developed significant nail changes such as dystrophy, onycholysis, paronychia, and pyogenic granulomas, with some requiring silver nitrate cauterization. Dermatological AEs typically resulted in the continued use of amivantamab at 67–75% of the original dose.

## 3. Teliso-V

Teliso-V (Telisotuzumab vedotin) is a first-in-class antibody–drug conjugate (ADC) designed to target c-Met-expressing tumor cells. It is composed of the anti-c-Met humanized monoclonal antibody ABT-700, linked to the cytotoxic agent monomethyl auristatin E (MMAE). Teliso-V specifically binds to c-Met with high affinity, facilitating the targeted delivery of MMAE directly to tumor cells. The first in-human phase 1 trial (NCT02099058) evaluated Teliso-V in advanced NSCLC with c-MET overexpression tumors (c-Met-positive; immunohistochemistry membrane H-score ≥ 150) that progressed despite standard therapy [39]. Out of sixteen patients treated with Teliso-V 2.4 to 3.0 mg/kg, three achieved partial response, with a median response duration of 4.8 months and mPFS of 5.7 months.

The same trial (NCT02099058) also studied Teliso-V in combination with other drugs. First, the trial evaluated Teliso-V plus osimertinib, an EGFR TKI that is the standard of care for EGFRm NSCLC, after previous failure with osimertinib in patients with c-MET overexpressing EGFRm NSCLC [40]. The ORR was 56% (*n* = 18), with similar response rates for both a high (≥50%) and intermediate (25–49%) MET expression. Second, Teliso-V plus erlotinib, another EGFR TKI, was evaluated in patients with c-MET-positive NSCLC (confirmed histology [H]-score ≥ 150) [41]. For all efficacy evaluable patients (*n* = 36), the mPFS was 5.9 months, including results from EGFRm and EGFR-WT. Specifically for the EGFRm cohort, the ORR was 32.1% (*n* = 28). Out of the EGFRm patients, those who were high in c-MET (H-score ≥ 250, *n* = 15) had an ORR of 52.6%. T790M is a common mutation for acquired resistance to EGFR TKIs. The mPFS for non-T790M patients was higher at 6.8 months than for T790M patients at 3.7 months. Finally, Teliso-V and nivolumab, an immunotherapy PD-L1 agent, were combined [42]. The ORR was 7.4%, with two patients (both PD-L1+- and c-MET-positive) having partial response (*n* = 27). The overall mPFS was 7.2 months. Due to limited antitumor activity, Teliso-V plus nivolumab was not pursued further.

The Phase 2 Luminosity (NCT03539536) trial aimed to evaluate the efficacy of Teliso-V in a variety of c-MET overexpressing (OE) NSCLC populations [43]. The ORR was 28.6% in c-MET OE non-squamous (NSQ) EGFR-WT NSCLC (*n* = 172), with a higher response rate for c-MET-high patients at 34.6%. The mDOR was 8.3 months (elevated in c-MET-high patients at 9.0 months). The mOS was 14.5 months, with little variation between high and intermediate c-MET expressors. Efficacy was only modest for c-MET OE NSQ EGFRm NSCLC (*n* = 43), with an ORR of only 11.6% [56]. Similarly, c-MET OE squamous (SQ) NSCLC had low efficacy at 11.1% (*n* = 27) [56]. Toxicities of note include pneumonitis (7.6%), peripheral sensory neuropathy (7.0%), and peripheral edema (16%). Overall, Teliso-V had durable responses in c-MET-overexpressing NSQ EGFR-WT NSCLC.

An ongoing phase 3 trial, TeliMET NSCLC-01 (NCT04928846), is comparing Teliso-V monotherapy versus docetaxel in c-MET-overexpressing EGFR-WT NSQ NSCLC that had previously progressed on therapy [44]. Additionally, another ongoing phase 2 trial (NCT05513703) is evaluating Teliso-V monotherapy in previously untreated MET Amp NSQ NSCLC with no targetable mutations (EGFR, ALK, ROS1, BRAF-WT).

## 4. Emibetuzumab

Emibetuzumab is a humanized immunoglobulin G4 monoclonal bivalent MET antibody that inhibits both ligand-dependent and ligand-independent HGF/MET signaling. The first-in-human phase 1 study (NCT01287546) evaluated emibetuzumab monotherapy versus emibetuzumab plus erlotinib (an EGFR TKI) in patients with advanced cancer (NSCLC specifically: *n* = 4 versus 14, respectively) [45]. Three durable partial responses were observed: one with emibetuzumab (700 mg) alone, and two with the combination of emibetuzumab and erlotinib (700 mg and 2000 mg). Notably, both responders to the combination therapy had previously progressed on erlotinib alone exhibited MET protein tumor expression and had EGFRex19 deletion mutation.

Due to the results from the phase 1 trial, another phase 2 trial (NCT01897480) evaluated the same drug combination: emibetuzumab plus erlotinib (*n* = 71) versus erlotinib monotherapy (*n* = 70) in MET-positive metastatic NSCLC with acquired resistance to erlotinib [46,47]. The mPFS for patients treated with the combination of emibetuzumab and erlotinib (EMI + E) was 9.3 months, compared to 9.5 months for those treated with erlotinib alone (E). Notably, patients with high MET expression (MET3+ expression in ≥90% of tumor cells; *n* = 24) revealed a significant improvement in PFS, with EMI + E showing a median PFS of 20.7 months compared to 5.4 months for E, suggesting that high-MET patients may receive clinically meaningful benefits from emibetuzumab and erlotinib.

Similarly, emibetuzumab monotherapy versus emibetuzumab plus erlotinib was also evaluated in a different phase 2 study in patients with acquired resistance to erlotinib (NCT01900652) [48]. Acquired resistance to erlotinib in MET diagnostic (+) patients was not reversed by emibetuzumab plus erlotinib or emibetuzumab monotherapy. However, a subset of patients experienced clinical benefits from the treatments. Consequently, there have been no further ongoing studies on emibetuzumab in the EGFR-mutated NSCLC space.

Another phase 1b/2 study combined emibetuzumab plus ramucirumab, a monoclonal anti-VEGFR-2 antibody targeting angiogenesis, in patients with advanced cancer (*n* = 15 NSCLC) (NCT02082210) [49]. The mPFS was 6.6 months for NSCLC patients. One patient had partial response (7%), and twelve had stable disease (80%), which was not a compelling antitumor response so there was no further investigation.

## 5. Other Novel c-MET Therapies

Onartuzumab is a monoclonal antibody that targets and binds to the extracellular domain of the MET receptor. A phase 2 trial (NCT00854308) compared onartuzumab plus erlotininb versus placebo plus erlotinib (EGFR TKI) in intent-to-treat (ITT) and MET-positive populations for recurrent NSCLC [50]. In the ITT population (*n* = 137), there was no improvement in mPFS or mOS. However, MET-positive patients (*n* = 66) treated with erlotinib plus onartuzumab showed improvements in both mPFS and mOS. On the other hand, MET-negative patients treated with the same erlotinib plus onartuzumab combination had worse clinical outcomes, emphasizing the importance of precise patient selection.

Based on the results, the phase 3 METLung trial (NCT01456325) analyzed the same drug combination, onartuzumab plus erlotininb versus erlotinib, in MET-positive patients whose disease progressed after platinum chemotherapy [51]. However, in this larger-scale study, onartuzumab plus erlotininb did not improve clinical outcomes versus erlotinib monotherapy (mOS of 6.8 versus 9.1, respectively). As a result, other trials involving onartuzumab plus erlotininb, such as a phase 2 study evaluating the efficacy of first-line onartuzumab plus erlotininb in MET-positive and EGFRm NSCLC, were also terminated [57]. Other trials studying onartuzumab plus first-line platinum chemotherapy in both advanced squamous and non-squamous NSCLC similarly showed a lack of clinical activity [58,59].

Sym015 is a novel MET antibody mixture of two humanized antibodies that induces MET degradation through a distinct mechanism that offers superior specificity compared to tyrosine kinase inhibitors (TKIs) [52]. In a phase 1/2a trial (NCT02648724), patients with MET exon 14 mutation (*n* = 12) or MET amplification (*n* = 8) were enrolled. Of the 20 patients, 5 had partial response (ORR 25%, 3/12 Ex14, 2/8 amp) and 11 had stable disease. For the ten patients that were MET TKI-naïve (3 Ex14, 7 amp), the ORR was 50%, and the DCR was 100%. mPFS was 5.5 months overall, with slight elevation in MET TKI-naïve patients compared to MET TKI pre-treated patients at 6.5 months versus 5.4 months. It was also well tolerated, as no patients discontinued or died due to a treatment-related adverse event; the most common treatment-related adverse events were fatigue (13.3%) and peripheral edema (11.1%).

REGN5093-M114 is an antibody–drug conjugate featuring bispecific MET x MET antibody and a novel linker-payload (M114) that carries the maytansine derivative M24, a potent inhibitor of microtubule assembly. Preclinical studies demonstrated significant antitumor activity in MET-overexpressing tumors compared to MET TKI, with response to REGN5093-M114 regardless of the MET gene copy number, thus presenting a promising candidate for overcoming functional MET pathway blockade [60]. Preliminary results demonstrated that 6/36 patients demonstrated partial response (5 previously had anti-PD-1 treatment) [61]. AEs (≥grade 3) occurred in 25% of cases, with pneumonia and pulmonary embolism each occurring in two patients. There is an ongoing phase 1/2 first in-human study (NCT04982224) evaluating the drug in MET-overexpressing NSCLC with no approved therapies expected to confer clinical benefit [53].

AZD9592 is a bispecific ADC designed to deliver a topoisomerase I inhibitor targeting EGFR and c-MET with a monovalent bispecific IgG platform. Preclinical studies in NSCLC models demonstrated significant tumor growth inhibition (TGI) in 73% of EGFRm NSCLC models, showcasing effectiveness in tumor targeting [62]. Additionally, the association between SLFN11 expression and treatment response highlights a potential biomarker for predicting sensitivity to AZD9592. An ongoing phase 1 clinical trial is being conducted with AZD9592, focusing on advanced solid malignancies, including NSCLC [63].

RC108 is another ADC composed of a targeted MET monoclonal antibody coupled to antimicrotubule drug monomethyl auristatin E by cleavable valine–citrulline. A phase 1 clinical trial is ongoing on c-MET-positive advanced malignant solid tumors [64].

## 6. Discussion

The exploration of anti-MET antibody drugs for NSCLC is a promising field in targeted cancer therapy, especially in overcoming resistance mechanisms associated with EGFR mutations. MET dysregulation, including protein overexpression, MET exon 14 skip mutation, and gene amplification, plays a crucial role in NSCLC pathogenesis and resistance to EGFR TKIs. The MET receptor’s involvement in diverse signaling pathways, tumor growth, and metastasis underscores its importance as a biomarker and therapeutic target.

Despite the significant role of MET in cancer progression, the clinical efficacy of MET-targeted therapies in NSCLC has been limited, partly due to the lack of effective predictive biomarkers [65]. Several biomarkers have been explored in selecting patients for MET-targeted clinical trials, including MET overexpression by IHC, gene copy number (GCN) by FISH and NGS, the MET/CEP7 ratio by FISH, and METex14 mutation by NGS. The METex14 mutation remains the most validated biomarker, leading to the approval of capmatinib for MET-altered NSCLC. MET amplification has also shown promise as a predictive biomarker, especially in patients with EGFR inhibitor resistance [65]. The ORR for patients with MET amplification varies significantly depending on the biomarker used. For patients with a MET/CEP7 ratio ≥2.0, the ORR ranges from 33% to 67%. In contrast, MET GCN ≥6 predicts a broader ORR range of 16% to 67%. Thus, MET amplification using the FISH MET/CEP7 ratio may be considered a biomarker with superior predictive accuracy, potentially due to its ability to distinguish true MET amplification, which is more likely to result in oncogenic addiction, from polysomy [66,67,68]. Future clinical research is needed to confirm the efficacy of MET amplification as a reliable biomarker and to explore additional biomarkers that could enhance the selection of appropriate patients for MET-targeted therapies in NSCLC.

Amivantamab, a bispecific EGFR/MET antibody, has emerged as a promising therapy for NSCLC, particularly in patients with EGFR mutations such as exon 20 insertions and those with progression following osimertinib therapy. Its dual-targeting mechanism distinguishes it from traditional single-target therapies by addressing both EGFR-driven oncogenesis and MET-related resistance mechanisms. Results from various clinical trials underscore the potential of amivantamab, both as a monotherapy and in combination regimens, to provide substantial clinical benefits in NSCLC with EGFR mutations. Despite promising outcomes, the use of amivantamab is associated with several notable challenges, particularly regarding its toxicity profile. Infusion-related reactions (IRRs) present a significant clinical hurdle, with rates as high as 78% for intravenous administration, although recent studies suggest that subcutaneous administration may mitigate these reactions, reducing the IRR rate to 13%. Hematologic toxicities, such as neutropenia and anemia, are also a concern, particularly when amivantamab is used in combination with chemotherapy, highlighting the need for careful monitoring and supportive care during treatment. Future research should focus on strategies to mitigate adverse effects associated with MET inhibition, aiming to optimize the therapeutic window and clinical benefit from amivantamab.

Similar concerns are seen with other MET-targeted agents like Teliso-V, which has shown durable responses in high-MET-expressing NSCLC but is also associated with adverse events such as peripheral neuropathy and edema. When combined with erlotinib, Teliso-V demonstrated enhanced antitumor activity, although the risk of neuropathy was elevated. Emibetuzumab, another MET antibody, demonstrated limited improvement in progression-free survival when combined with erlotinib, but showed potential in patients with high MET expression. It was generally well tolerated, with common adverse events such as peripheral edema and mucositis.

Antibody-based agents, such as amivantamab, and MET TKIs like capmatinib and tepotinib, offer distinct approaches in targeting MET dysregulation in NSCLC. Antibody-based therapies, particularly bispecific antibodies, provide the advantage of simultaneously targeting multiple pathways, such as EGFR and MET, making them effective in overcoming resistance mechanisms that arise from EGFR mutations. These agents can deliver potent cytotoxic payloads directly to tumor cells, enhancing tumor specificity and reducing off-target effects. In contrast, MET TKIs, including capmatinib and tepotinib, primarily inhibit the tyrosine kinase activity of MET, effectively blocking downstream signaling pathways involved in tumor growth and survival.

One limitation of MET TKIs is the development of resistance. The main mechanism of resistance is secondary mutations in the MET gene, notably at residues Y1230 and D1228 [69]. These mutations alter the kinase domain, reducing the binding affinity of TKIs, which leads to diminished therapeutic efficacy. For example, Y1230 mutations interfere directly with the binding of Type I MET TKIs like capmatinib, while D1228 mutations can also lead to resistance across various TKIs [70,71]. Other resistance mechanisms are off target, such as KRAS amplifications, EGFR amplifications, HER2 amplifications, and acquired mutations in BRAF and KRAS [72]. Such resistance highlights the limitations of MET TKIs and the need for alternative therapeutic strategies. Antibody-based agents, such as bispecific antibodies and ADCs, offer alternative methods to overcome TKI resistance by targeting MET through distinct mechanisms that are less likely to be affected by the mutations that confer TKI resistance. These agents can also engage the immune system or deliver cytotoxic agents directly to the cancer cells, thereby bypassing the resistance mechanisms that undermine the effectiveness of MET TKIs.

The combination of antibody-based therapies and MET TKIs are a possibility. Many trials have previously combined chemotherapy or immunotherapy. Others have combined ADCs with EGFR TKIs, notably amivantamab and lazertinib (MARISOPA), which has been shown to be effective, receiving approval from the FDA as a first-line treatment for locally advanced/metastatic NSCLC with exon 19 deletion or exon 21 L858R substitution mutation in 2024 [38,54]. Despite the potential of MET TKI and ADC combinations, safety remains a concern, as both classes of drugs can cause overlapping toxicities, such as liver toxicity, hematologic effects, and gastrointestinal issues. Additionally, the risk of adverse effects like peripheral edema, commonly seen with MET-targeted therapies, could be exacerbated. Although the potential of combination therapy has been demonstrated, additional trials are necessary to determine optimal dosing strategies and to fully assess the safety and efficacy of this combination approach in MET-altered NSCLC.

The development of anti-MET antibody drugs signifies a crucial advancement in addressing resistance mechanisms in NSCLC. Reviewed clinical trials suggest that combination therapies and precise patient selection based on MET status are pivotal for maximizing efficacy. Future research should focus on refining biomarker-driven approaches to enhance the precision of MET-related biomarkers, exploring additional combinations with other targeted therapies, immunotherapies, and conventional treatments to overcome resistance and improve outcomes. Additionally, further research of the mechanisms of MET signaling and its interaction with other pathways is essential to develop more effective therapeutic strategies. In conclusion, while challenges remain, the progress in anti-MET antibody drug development holds substantial promise for improving the management and outcomes of NSCLC. Continued research and clinical trials will be instrumental in translating these therapies into improved NSCLC outcomes.

## Figures and Tables

**Table 1:** List of clinical trial results for MET antibody drugs.

Drug	Trial	Target Population	Results
Amivantamab	CHRYSALIS (NCT02609776)	EGFR Exon20ins (*n* = 81) progressed on chemotherapy [29]	ORR, 40% mDOR, 11.1 mo mPFS, 8.31 mo
Amivantamab plus lazertinib		EGFRm with progression on osimertinib but chemotherapy-naïve (*n* = 45) [30]	ORR, 36% mDOR, 9.6 mo mPFS, 4.9 mo
Amivantamab		MET positive with MetEx14 mutation (*n* = 36) [31]	ORR, 33.3% mPFS, 6.7 months
Amivantamab plus capmatinib	METalmark (NCT05488314)	MetEx14 mutation and MET-amplified (*n* = 161)	Ongoing
Amivantamab plus CT	PAPILLION (NCT04538664)	Ami-CT (*n* = 153) vs. CT alone (*n* = 155) as 1L for EGFR Exon20ins mutation [32]	Ami-CT vs. CT ORR, 73% vs. 47% mPFS, 11.4 vs. 6.7 mo
Amivantamab plus lazertinib plus CT	CHRYSALIS-2 (NCT04077463)	EGFRm with progression on or after EGFR TKI (*n* = 20) [33]	ORR, 50% mPFS, 14.0 months
Amivantamab plus lazertinib		post-chemo/post-osimertinib EGFRm Exon19del or L858R (*n* = 162; 50 efficacy evaluable) [34]	ORR, 36% Clinical benefit rate, 58%
Amivantamab plus lazertinib		Atypical non-exon 20 insertion EGFRm (*n* = 40) in post-afatinib and treatment-naïve [35]	PR in 60% patients ORR, 56.7%
Amivantamab plus lazertinib	PALOMA-3 (NCT05388669)	subcutaneous vs. intravenous administration in post-CT/post-osimertinib EGFRm (*n* = 418) [36]	ORR, 30% vs. 33% mPFS, 6.1 mo vs. 4.3 mo
Amivantamb plus lazertinib	MARIPOSA (NCT04487080)	Amivantamb plus lazertinib (*n* = 429) versus osimertinib (*n* = 429) as 1L EGFRm [37]	Ami-laz vs. osi ORR, 86% vs. 85% mDOR, 25.8 vs. 16.8 mo mPFS, 23.7 mo vs. 16.6 mo
Amivantamab–lazertinib–chemotherapy	MARIPOSA-2 (NCT04988295)	Ami-laz-CT vs. ami-CT versus CT alone (2:1:2 ratio, *n* = 657) in EGFRm after progression on osimertinib [38]	Ami-laz-CT vs. ami-CT vs. CT ORR, 63% vs. 64% vs. 36% mPFS, 8.2 vs. 8.3 vs. 4.2 mo
Teliso-V	NCT02099058	Advanced NSCLC with c-MET overexpression (*n* = 16) [39]	3/16 partial reponse DOR, 4.8 month mPFS, 5.7 month
Teliso-V plus osimertinib		c-MET overexpressing, EGFRm, after failure on previous osimertinib (*n* = 18) [40]	ORR, 56%
Teliso-V plus erlotinib		c-MET positive EGFRm (*n* = 26) and EGFR WT (*n* = 8) [41]	mPFS, 5.9 mo EGFRm: ORR, 32.1% Non-T790M: mPFS, 6.8 mo
Teliso-V plus nivolumab		c-MET positive EGFRm (*n* = 27) [42]	ORR, 7.4% mPFS, 7.2 month
Teliso-V	Luminosity (NCT03539536)	MET overexpressing (details elaborated on in text) [43]	NSQ EGFR-WT: ORR, 52.2% NSQ EGFRm: ORR, 11.6% SQ EGFR-WT: ORR, 11.1%
Teliso-V	TeliMET NSCLC-01 (NCT04928846)	Teliso-V vs. docetaxel in c-MET overexpressing, EGFR-WT, NSQ with progression [44]	Ongoing
Teliso-V	NCT05513703	Previously untreated MET Amp NSQ NSCLC with no targetable mutation	Results not published
Emibetuzumab plus erlotinib	NCT01287546	EM + ER vs. EM in NSCLC (*n* = 4 vs. 14, respectively) [45]	1/4 EM partial response 2/14 EM-ER partial response
Emibetuzumab plus erlotinib	NCT01897480	EM + ER (*n* = 71) vs. ER (*n* = 70) in MET-positive with erlotinib resistance [46,47]	EM-ER vs. ER mPFS 9.3 vs. 9.5 mo High MET expression: mPFS, 20.7 vs. 5.4 mo
Emibetuzumab plus erlotinib	NCT01900652	EM + ER vs. EM (*n* = 111, 3:1 ratio) with ER resistance [48]	EM-ER vs. EM ORR, 3.0% vs. 4.3%
Emibetuzumab plus ramucirumab	NCT02082210	Advanced NSCLC (*n* = 15) [49]	mPFS, 6.6 mo 1/15 partial response 12/15 stable disease
Onartuzumab plus erlotininb	NCT00854308	onartuzumab plus ER vs. ER in ITT (*n* = 137) and MET-pos (*n* = 66) [50]	Onartuzumab + ER vs. ER ORR, 5.8% vs. 4.4% mPFS, 2.2 vs. 2.6 mo
Onartuzumab plus erlotininb	METLung trial (NCT01456325)	onartuzumab plus ER vs. ER in MET-pos after chemo progression [51]	Onartuzumab + ER vs. ER mOS, 6.8 vs. 9.1 mo
Sym015	NCT02648724	MetEx14 (*n* = 12) or MET amp (*n* = 8) [52]	5/20 PR mPFS, 5.5 mo MET TKI naïve (*n* = 10): ORR, 50%, 6.5 mo
REGN5093-M114	NCT04982224	MET-overexpressing NSCLC with no approved therapies [53]	Ongoing

ORR: overall response rate; mDOR: median duration of response; mPFS: median progression-free survival; DOR: duration of response; PR: partial response; CT: chemotherapy; MetEx14: MET exon 14 skipping mutation; Ami: amivantamab; Laz: lazertinib; Osi: osimertinib; EGFRm: EGFR mutation, NSQ: non-squamous; SQ: squamous; EM: emibetuzumab; ER: erlotinib; TKI: tyrosine kinase inhibitor.

## Data Availability

Not applicable.
